# Anti‐inflammatory and anti‐insulin resistance activities of aqueous extract from *Anoectochilus burmannicus*

**DOI:** 10.1002/fsn3.416

**Published:** 2016-08-26

**Authors:** Phatcharaporn Budluang, Pornsiri Pitchakarn, Pisamai Ting, Piya Temviriyanukul, Ariyaphong Wongnoppawich, Arisa Imsumran

**Affiliations:** ^1^ Department of BiochemistryFaculty of MedicineChiang Mai UniversityMeungChiang MaiThailand; ^2^ Food and Nutritional Toxicology UnitInstitute of NutritionMahidol UniversitySalayaNakhon PathomThailand

**Keywords:** 3T3‐L1 adipocyte, *Anoectochilus burmannicus*, inflammation, insulin resistance, RAW264.7 macrophage

## Abstract

This study investigated biological activities including antioxidative stress, anti‐inflammation, and anti‐insulin resistance of *Anoectochilus burmannicus* aqueous extract (ABE). The results showed abilities of ABE to scavenging DPPH and ABTS free radicals in a dose‐dependent manner. Besides, ABE significantly reduced nitric oxide (NO) production in the lipopolysaccharide (LPS)‐treated RAW 264.7 via inhibition of mRNA and protein expressions of nitric oxide synthase (iNOS). The LPS‐induced mRNA expressions of cyclooxygenase‐2 (COX‐2) and interleukin 1β (IL‐1β) were suppressed by ABE. Moreover, ABE exerted anti‐insulin resistance activity as it significantly improved the glucose uptake in tumor necrosis factor (TNF)‐α treated 3T3‐L1 adipocytes. In addition, ABE at the concentration of up to 200 μg/mL was not toxic to human peripheral blood mononuclear cells (PBMCs) and did not induce mutations. Finally, the results of our study suggest the potential use of *A. burmannicus* as anti‐inflammatory, anti‐insulin resistance agents, or food supplement for prevention of chronic diseases.

## 1 Introduction

Chronic inflammation is widely known to be complicated in pathogenesis of various diseases such as Type 2 diabetes mellitus, obesity, metabolic syndrome (Esser, Legrand‐Poels, Piette, Scheen, & Paquot, 2014; Xu et al., 2003), and cancer (Chen, Alvero, Silasi, & Mor, 2007). Several physical and chemical stimulants are factors involved in the inflammatory process such as free radicals which cause oxidative stress (Li & Wang, 2011). During an inflammatory response, excessively high levels of oxidative stress often result in cellular damage by initiating chemical chain reactions such as lipid peroxidation or the oxidation of DNA and proteins leading to cellular dysfunction (Agarwal, Saleh, & Bedaiwy, 2003; Lobo, Patil, Phatak, & Chandra, 2010) and cell death (Lefebvre et al., 2002).

Macrophage plays important roles in the autoregulatory loop of the inflammatory process (Shibata et al., 1998). Lipopolysaccharide (LPS) can activate toll‐like receptor 4 (TLR4) in macrophages to induce the expression of many inflammatory genes causing the production of several inflammatory mediators such as nitric oxide (NO), prostaglandins (PGs), and many proinflammatory enzymes (Creely et al., 2007). Cyclooxygenase‐2 (COX‐2) and inducible nitric oxide synthase (iNOS) are directly responsible for elevated levels of NO and PGs, respectively, leading to promotion of pathological inflammation (Cho, Cho, & Song, 2005; Huang et al., 2013). Moreover, NO has effect on COX‐2 activity (Li & Wang, 2011; Salvemini et al., 1993).

LPS‐stimulated macrophages mediate the inflammatory response by releasing not only NO but also a variety of proinflammatory cytokines, such as tumor necrosis factor (TNF‐α), interleukin 1β (IL‐1β), and interleukin‐6 (IL‐6), which are related to pathogenesis diseases (Popa, Netea, van Riel, van der Meer, & Stalenhoef, 2007). These cytokines especially TNF‐α could induce insulin resistance in adipocytes (Neels & Olefsky, 2006). TNF‐α causes a downregulation of many protein expressions in adipocytes such as insulin receptor (IR) (Engelman, Berg, Lewis, Lisanti, & Scherer, 2000) and GLUT4 (Stephens, Lee, & Pilch, 1997) leading to a decrease in glucose uptake upon stimulation by insulin. In addition, TNF‐α activates an activator protein‐1 (AP‐1) transcription factor (Westwick, Weitzel, Minden, Karin, & Brenner, 1994; Wu et al., 2011), nuclear factor kappa B (NF‐κB) (Popa et al., 2007; Schutze, Wiegmann, Machleidt, & Kronke, 1995), interferon regulatory factor (IRF) (Vila‐del Sol, Punzon, & Fresno, 2008), and protein kinase R (PKR) (Meusel, Kehoe, & Imani, 2002) to regulate the expression of other cytokine genes. In animal model, several studies showed the crosslink between signaling pathways of inflammation and insulin resistance. For example, attenuated TNF‐α and TNF‐α receptor, and JNK signaling in high‐fat diet‐treated mice could reduce inflammation markers and blood sugar levels (Uysal, Wiesbrock, Marino, & Hotamisligil, 1997), and elevated the sensitivity of insulin signaling pathway (Hotamisligil, 1999; Nieto‐Vazquez et al., 2008). Therefore, reduction in inflammation by inhibiting proinflammatory cytokine production would be an alternative way to improve the insulin sensitivity of adipocytes and induce the cellular glucose uptake.

In recent years the researchers have tried to substantiate anti‐inflammatory and anti‐insulin resistance properties of many natural products. It is noteworthy that effective therapeutic agents on controlling a variety of inflammatory diseases mostly target macrophages and their products (Fujiwara & Kobayashi, 2005). Many studies have extensively focused on anti‐inflammation and metabolic syndrome. For example, anti‐TNF‐α blockade can improve insulin resistance in rheumatoid arthritis patients (Gonzalez‐Gay et al., 2006). Interleukin‐1‐receptor antagonist has been reported to increase insulin sensitivity in type 2 diabetes mellitus patient (Akash, Shen, Rehman, & Chen, 2012). Thus, studies of anti‐inflammation have become a main target for prevention and treatment of obesity‐related insulin resistance.

Herbal medicine has been widely used as dietary supplement among Thai people to maintain or improve their health. Several Thai traditional plants such as ginger (Grzanna, Lindmark, & Frondoza, 2005), longan (Ho, Hwang, Shen, & Lin, 2007; Wang, Tang, Chiu, & Huang, 2012), and *Gymnema inodorum* (Shimizu et al., 2001) have been reported for their antioxidant and anti‐inflammatory activities. Thus, they have potential value in treating various diseases including diseases involving inflammation.

*Anoectochilus* genus such as *Anoectochilus roxburghii, Anoectochilus formosanus, Anoectochilus elwesii,* and *Anoectochilus setaceus* are used widespread in China, Vietnam, Sri Lanka, and Taiwan because of their medicinal properties (Yang, Wu, Lu, & Lin, 2013). They have remarkable curative effects of clearing heat and cooling blood, removing dampness, detoxification, antioxidative stress (Shih, Wu, & Lin, 2002; Wang et al., 2005), and anti‐inflammation (Hsieh, Hsiao, & Lin, 2010). Previous studies reported that kinsenoside isolated from *Anoectochilus roxburghii* and *Anoectochilus formosan*us provide beneficial effects on hepatoprotective (Wang et al., 2005), antihyperlipolysis activities (Du et al., 2001). It also reduces blood glucose (Zhang, Cai, Ruan, Pi, & Wu, 2007; Zhang, Liu, Liu, Li, & Yi, 2015), modulaties the allergic response, and exerts anti‐inflammation in both the LPS‐induced inflammation in macrophages and endotoxin‐shocked mice (Hsiao, Wu, Lin, & Lin, 2011).

In this study, we focused on biological effects of *A. burmannicus*, which is found in the northern part of Thailand. We aimed to determine whether *A. burmannicus* aqueous extract exerts antioxidative stress, anti‐inflammation, and anti‐insulin resistance. Aqueous extract of ABE was prepared and its phenolic content determined. Biological properties were examined including antioxidative stress and anti‐inflammation in LPS‐induced RAW 264.7 macrophage cells and anti‐insulin resistance in TNF‐α‐induced 3T3‐L1 adipocytes. The knowledge from this study would provide supportive evidences for implementation and development of this plant as an alternative medicine or functional food in order to prevent and/or treat chronic diseases including type 2 diabetes mellitus which could be caused by inflammation.

## 2 Materials and Methods

### 2.1 Preparation of plant extracts

Plant materials and extraction were cultivated by tissues culture engineering (Queen Sirikit Botanic Garden, Chiang Mai, Thailand). Dried whole plant of *A. burmannicus* was soaked in diH_2_O and subjected to autoclave at 121°C for 30 min according to the method of Kim & Jang, (2011) with slight modifications. Then, the aqueous fraction was filtered and lyophilized to obtain crude ABE powder.

### 2.2 Phytochemical screening

#### 2.2.1 Determination of total phenolic compounds

Total phenolic content of ABE was determined according to the method of by Subedi et al., (2014) with slight modifications. Briefly, 300 μL of the extracts was added to 400 μL of 10% equivalent Folin–Ciocalteu reagent and incubated for 3 min in dark, room temperature. Then 300 μL of 7.5% equivalent/L Na_2_CO_3_ was added and the mixture was allowed to stand for 20 min in dark, after which absorbance readings at 765 nm was measured using a spectrophotometer. The phenolic content was calculated using the linear equation based on the calibration curve and expressed as the total phenolic contents equivalence (CE)/g dry weight.

#### 2.2.2 Determination of phenolic derivatives content in ABE

Phenolic compounds were determined by HPLC using a C18 column (250 mm × 4.6 mm, 5 μm). Gradient elution was performed using two solvents; A (0.1% trifluoroacetic acid in water) and B (100% methanol) for determining phenolic compounds. Ten microliters of the 10 mg of ABE was dissolved in 1 mL of diH_2_O and injected into the column with a flow rate 1.0 ml/min and detected at 280 and 325 nm. Peak area and retention time of the extract sample were compared with standard curves of various concentrations of standard catechin, chlorogenic acid, coumaric acid, ferrulic acid, gallic acid, hydroxybenzoic acid, protocatecheuic acid, and vanillic acid.

### 2.3 Determination of antioxidant activity

#### 2.3.1 ABTS radical scavenging assay

The ABTS^+^ scavenging ability was determined according to the method of Jatinder Kumar et al., (2014). A mixture of 7 mmol/L ABTS^+^ stock solution with 2.45 mmol/L potassium persulfate (1:1, v/v) was left in the dark at room temperature for 12–16 hr until the reaction was complete and the absorbance was stable. The ABTS^+^ reagent was diluted with 95% ethanol to an absorbance of 0.700 ± 0.02 at 734 nm (Microplate reader Synergy Hybrid Reader, BioTek). An aliquot (10 μL) of each sample was mixed with 990 μL ABTS^+^ reagent and the absorbance was read at 734 nm after 6 min at 30°C in the dark. A reference standard was Trolox. The antioxidant activity was defined as the IC_50_ required to scavenging ABTS^+^ radicals by 50%, which was calculated from a log‐dose inhibition curve.

#### 2.3.2 DPPH radical scavenging assay

DPPH radical scavenging activity was evaluated using the following method from a previous study (Wang, Wang, & Yih, 2008). Twenty microliters of ABE solution were mixed with 180 μL of diphenyl‐p‐picrylhydrazyl radical (DPPH) in ethanol and 20 μL of different concentrations of sample and then incubated for 20 min at room temperature with light protection. The remaining DPPH was measured by colorimetric at 517 nm using a microplate reader (Synergy Hybrid Reader). The antioxidant activity of sample was shown as the IC_50_ required to scavenging DPPH radicals by 50%, which was calculated from an inhibition curve.

### 2.4 Genotoxicity assessment of ABE in *Drosophila melanogaster*

The somatic mutation and recombination test (SMART) was initially demonstrated by Graf et al., (1984). Briefly, males of *mwh/mwh* and females of *ORR;flr* ^*3*^ */In(3LR) TM3, ri p* ^*p*^ *sep l(3)89Aa bx* ^*34e*^ *e Bd* ^*S*^ *, Ser* were mated to produce transheterozygous larvae (*mwh flr* ^*+*^ */mwh TM3*). Then, 100 transheterozygous larvae were cultured on standard medium containing water (negative control), ABE (500 μg/ml), or 20 mmol/L urethane (positive control). The phenotypes on the wings were scored as previously reported (Laohavechvanich, Kangsadalampai, Tirawanchai, & Ketterman, 2006). Lastly, statistical significance was calculated according to Frei & Wurgler (1988).

### 2.5 Cell lines and cell culture

RAW 264.7 macrophage‐like cell line was obtained from CLS‐Cell Lines Service, Germany. The cell line was cultured in an ultralow attachment culture dish in DMEM with l‐glutamine supplemented with 10% heat‐inactivated fetal bovine serum (FBS) and 1% penicillin/streptomycin solution and maintained at 37°C in a 5% CO_2_ humidified atmosphere CO_2_ incubator (Thermo Scientific). The cells were subjected to the experiments or subculture when they reached confluence of 80%. 3T3‐L1 adipocyte cells from ATCC (American Type Culture Collection) were cultured in DMEM with l‐glutamine supplemented with 10% FBS and 1% penicillin/streptomycin solution and maintained at 37°C in a 5% CO_2_ humidified atmosphere CO_2_ incubator. After reaching confluence on day 2, 3T3‐L1 preadipocytes were differentiated by inducing with 0.5 mmol/L 3‐isobutyl‐1‐metylxanthine (IBMX), 0.5 μg/mL dexamethasone, 5 μg/mL insulin, and 10% fetal ovine serum for 72 hr, followed by another 72 hr in the same medium without IBMX and dexamethasone. Complete cell differentiation was obtained by incubating the cells in DMEM containing 10% FBS for 7–14 days. Human peripheral blood mononuclear cells (PBMCs) were isolated using Ficoll‐hypaque. The mononuclear cells were carefully collected and rinsed twice with ice‐cold phosphate‐buffered saline (PBS) pH 7.4 and resuspended in fresh RPMI medium.

### 2.6 (2‐(2‐methoxy‐4‐nitrophenyl)‐3‐(4‐nitrophenyl)‐5‐(2,4‐disulfophenyl)‐2H‐tetrazolium (WST‐1) assay

Macrophage cells (RAW 264.7), 3T3‐L1 adipocytes, and human PBMCs were seeded in 96‐well culture plates at 1 × 10^4^, 5 × 10^3^, and 8 × 10^4^ cells/well, respectively. Then, they were treated with various doses of ABE for 24 hr. At the end of treatment, cell supernatant was removed and then 100 μL of WST‐1 reagent solution was added to each well and incubated for 2 hr at 37°C. The WST‐1 soluble formazan was measured using a microplate reader (Synergy Hybrid Reader) at 440 nm.

### 2.7 Treatment protocol of RAW 264.7 macrophages

RAW 264.7 macrophage cells were seeded in plates or dish for 24 hr. Then, medium was removed and replaced fresh medium with or without different concentrations of the ABE (0–200 μg/ml). After 2 hr of the incubation, 1 μg/mL of LPS was added and further incubated for 24 hr. After the treatment, cell‐free culture medium or cell pellets were collected for further studies.

### 2.8 Determination of NO production

After the treatment, the cell‐free culture medium was collected to measure the amount of nitric oxide using Griess reagent (Sigma Aldrich) as described in the manufacturer's protocol. The amount of nitrite present in the samples was calculated by means of a standard curve generated using serial dilutions of NaNO_2_ in fresh culture medium.

### 2.9 Cytokine production determinations

The levels of IL‐6 and TNF‐α in the culture medium of the treated cells were determined by sandwich Enzyme Link Immuno‐Sorbent Assay (BioLegend's ELISA MAXTM Deluxe Set, CA). Briefly, 100 μL of supernatant after treatment was used and assayed according the manufacturer's protocol for the relevant ELISA kit.

### 2.10 Anti‐insulin resistance assay

To induce insulin resistance, mature 3T3‐L1 adipocytes were treated with 50 ng/mL of TNF‐α for 24 hr, after that the cells were washed with phosphate‐buffered saline (PBS) and further treated with various concentrations of ABE for 24 hr. Next, they were washed with PBS and incubated with low glucose DMEM for 3 hr at 37°C. The medium was then replaced by 1 mg/mL bovine serum albumin (BSA) containing 100 mmol/L 2‐[N‐(7‐nitrobenz‐2‐oxa‐1, 3‐diazol‐4‐yl) amino]‐2‐deoxy‐glucose (2‐NBDG‐glucose) and 100 nmol/L insulin, and incubated for 1 hr at 37°C. Fluorescence intensity of intracellular 2‐NBDG‐glucose was measured at λex = 485 nm and λem = 535 nm using a microplate reader.

### 2.11 Immunoblot analysis

After the treatment with cytokine, RAW 264.7 cells were collected and lysed with RIPA buffer containing protease inhibitors. The protein samples were subjected to 10% SDS‐PAGE and transferred to nitrocellulose membranes. The membranes were blocked with 3% BSA in tween‐TBS at 4°C, and then probed with primary antibodies specific to COX‐2 (Cell signaling, USA), iNOS (Merck, USA), or β‐actin (Sigma, USA) overnight. The membrane was washed five times with 0.015% tween‐TBS for 30 min, and was incubated with horseradish peroxidase (HRP)‐conjugated IgG secondary antibody for 2 hr at room temperature. An enhanced chemiluminescence (ECL) system was utilized to detect the HRP signal. Equal protein loading was evaluated in each membrane, which was stripped and reprobed with anti‐β‐actin.

### 2.12 Reverse Transcription‐quantitative Polymerase Chain Reaction (RT‐qPCR) of inflammatory mediators mRNA

The mRNA expressions of TNF‐α, IL‐1β, IL‐6, iNOS, and COX‐2 were measured by reverse transcription (RT) quantitative PCR. RAW 264.7 macrophage cells were plated overnight in 6‐well plate and incubated with ABE for 2 hr, and then stimulated with LPS (1 μg/mL) for 24 hr. Total RNA was extracted using Trizol reagent (Invitrogen). First‐strand cDNA was synthesized using 1 μg of total RNA with oligo (deoxythymidine) primers and Superscript II reverse transcriptase (Invitrogen). The target cDNA was subsequently used as the template for RT‐qPCR amplification using THUNDERBIRD^™^ SYBR^®^ qPCR Mix (Toyobo) and primers for IL‐1β (sense, 5′‐ AAGGGCTGCTTCCCAACCTTTGAC‐3′; antisense, 5′‐ATACTGCCTGCCTGAAGCTCTTGT‐3′), IL‐6 (sense, 5′‐ CCAGAAACCGCTATGAAGTTCC3′; antisense, 5′‐ TCACCAGCATCAGTCCCAG‐3′), TNF‐α (sense, 5′‐ CTCCAGGCGGTGCCTATGT3′; antisense, 5′‐ GAAGAGCGTGGTGGCCC‐3′), COX‐2 (sense, 5′‐ CCGAGGTGTATGTATGAG‐3′; 5′‐ TGGGTAAGTATGTAGTGC‐3′), iNOS (sense, 5′‐ CTTTGGTGCTGTATTTCC‐3′; antisense, 5′‐ TGTGACCTCAGATAATGC‐3′), and GAPDH (sense, 5′‐ TGGCAAAGTGGAGATTGTTGCC‐3′; antisense, 5′‐ AAGATGGTGATGGGCTTCCCG‐3′). The reaction conditions were followed by 40 cycles of 70°C for 5 min, 4°C for 1 min, and 42°C for 60 min. The level of target cDNA was normalized by the expression of GAPDH and then measured as relative expression to the LPS‐treated control.

### 2.13 Statistical analysis

All values were given as mean ± standard derivation (X ± SD) from triplicate samples of three independent experiments. Overall differences among the treatment groups were determined using one‐way analysis of variance (ANOVA) by Prism 5.0 software. *p* < 0.05 is regarded as significance.

## 3 Results

### 3.1 Phenolic acid composition of the ABE

The water extraction of *A. burmannicus* provided yield of ABE at 16.79%. Total phenolic content of ABE was 19.09 ± 1.18 mg CE/g. To establish the HPLC fingerprint chromatogram for the quality control of ABE, eight phenolic acids were quantitatively analyzed. It was found that ABE contains vanillic acid (1.647 ± 0.013 mg/g extract), ferulic acid (0.169 ± 0.008 mg/g extract), chlorogenic acid (0.612 ± 0.029 mg/g extract), and coumaric acid (0.058 ± 0.001 mg/g extract).

### 3.2 Effect of ABE on scavenging of DPPH and ABTS radicals

**Figure 1:**
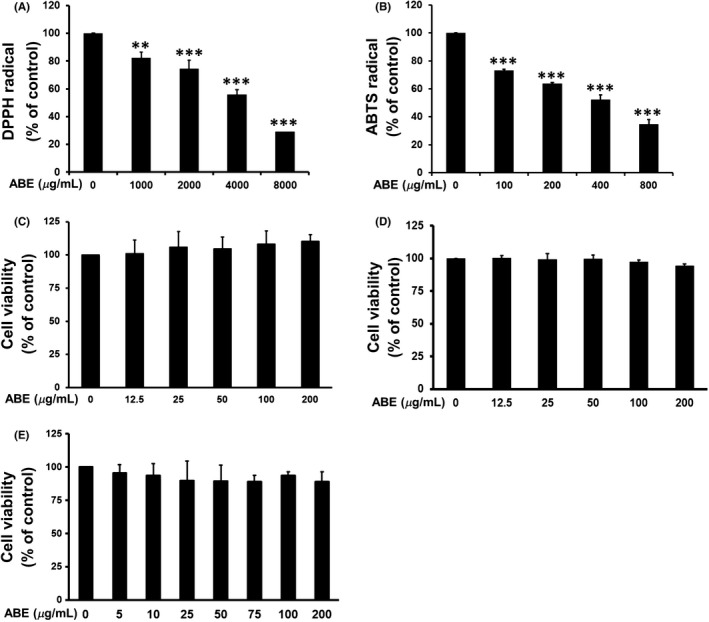
Free radical scavenging activity of ABE determined by DPPH (A) and ABTS (B) assays. Effect of ABE on cell viability of murine macrophage raw 264.7 cells (C), murine mature 3T3‐L1 adipocytes (D), and human PBMCs (E). ** *p*<0.01, *** *p*<0.001 vs. control (non‐treated group). ABE, *Anoectochilus burmannicus* aqueous extract

Antioxidant activities of ABE were determined using DPPH and ABTS assays as shown in Figure 1. ABE had ability to scavenge DPPH free radical up to 70% (*p* < 0.05) in a dose‐dependent manner. The 50% inhibition concentration (IC_50_) was 3.39 ± 0.23 mg/mL (Fig. 1A). The ABTS assay system is a decolorization technique in which the radical is generated directly and stable. In this assay, the ABE fraction exhibited the suppressive effect on ABTS radical with an IC_50_ value of 0.38 ± 0.02 mg/mL (Fig. 1B). These results suggested that ABE exerted antioxidant property which likely provides protective effect on oxidative stress related to inflammation.

### 3.3 Effect of ABE on genome stability of *Drosophila melanogaster*

To assess the genotoxic properties of ABE, the *in vivo* assay involving *Drosophila* wing spot test (SMART) was employed. The assay is capable of detecting several types of DNA mutations, for instance, point mutations, nucleotide deletions, DNA breaks, and failure of recombination (Graf, Abraham, Guzman‐Rincon, & Wurgler, 1998). As shown in Table 1, 20 mmol/L urethane statistically induced DNA mutations (total spot), which is in line with a formerly report (Laohavechvanich et al., 2006). The negative control together with ABE did not provoke any type of mutations implying that ABE was not genotoxic, although the used dose of ABE (500 μg/mL) was even higher than that of cytotoxicity assay (Fig. 1C–E).

**Table 1:** Effect of ABE on *Drosophila* genome

Experiment	Treatment		Spot per wing (Number of spots from 40 wings)^a^			
	Sample	Concentration	Small single (m = 2)	Large single (m = 5)	Twin spot (m = 5)	Total spot (m = 2)
1	Negative control	—	0.30 (12)	0.03 (1)	0 (0)	0.33 (13)
	Urethane	20 mmol/L	8.10 (324)+	2.08 (83)+	1.10 (44)+	11.28 (451)+
	ABE	500 μg/ml	0.20 (8)−	0.08 (3)i	0.05 (2)i	0.33 (13)i
2	Negative control	—	0.43 (17)	0.10 (4)	0.03 (1)	0.55 (22)
	Urethane	20 mmol/L	18.85 (754)+	6.88 (275)+	3.03 (121)+	28.75 (1150)+
	ABE	500 μg/ml	0.28 (11) −	0.05 (2) −	0.05 (2)i	0.38 (15) −

a Statistical diagnoses using estimation of spot frequencies and confidence limits according to Frei and Würgler (1988) for comparison with deionized water; +, Positive; −, Negative; i, Inconclusive.

Probability levels: *α* = *β* = 0.05. One‐sided statistical test “m” is an increased mutation frequency compared with the spontaneous frequency (m times). ABE, *Anoectochilus burmannicus* aqueous extract.

### 3.4 Effect of ABE on cell viability of normal human PBMCs, RAW 264.7 macrophages, and 3T3‐L1 adipocytes

To assess the cytotoxicity, normal human PBMCs, macrophages RAW 264.7, and 3T3‐L1 adipocytes were treated with different dosages of ABE (0–200 μg/mL) for 24 hr. ABE at the concentrations up to 200 μg/mL did not affect the viability of these cells, as observed by WST‐1 assay (Fig. 1C–E). Nontoxic doses of ABE (0–200 μg/mL) were then chosen in the further experiments.

### 3.5 Effect of ABE on NO production in LPS‐stimulated RAW 264.7 macrophages

**Figure 2:**
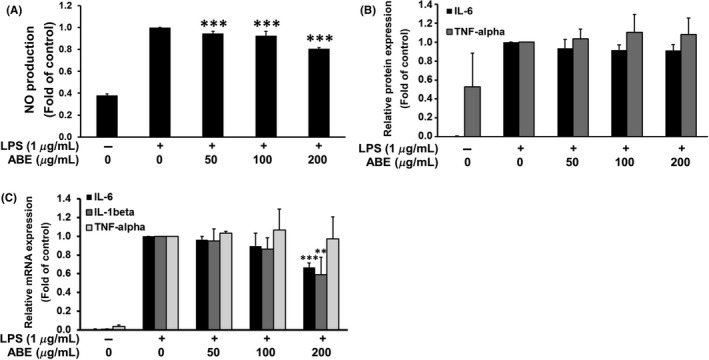
Effects of ABE on nitric oxide (NO) production (A), on protein expression of tumor necrosis factor (TNF‐α), and inerleukin 6 (IL‐6) (B) and on mRNA expression of IL‐6, IL1‐β, and TNF‐α (C). ** *p*<0.01, *** *p*<0.001 vs. LPS‐treated group ABE, *Anoectochilus burmannicus* aqueous extract

To assess the anti‐inflammatory activity *in vitro*, we determined the inhibitory effect of ABE on NO production in LPS‐treated RAW 264.7 cells. The NO release was measured as the accumulation of nitrite in the culture supernatant. Significantly, ABE inhibited the LPS‐induced nitrite production in a dose‐dependent manner by 10–20% as compared to LPS‐treated group (Fig. 2A).

### 3.6 Effect of ABE on LPS‐induced TNF‐α, IL‐1β, and IL‐6 productions in RAW 264.7 macrophages

Proinflammatory cytokines including IL‐1β, IL‐6, and TNF‐α play major roles in the inflammation cascades (Lee et al., 2014). Under basal condition, macrophages produced low levels of IL‐1β, TNF‐α, or IL‐6, whereas LPS could induce the expression of these cytokines. ABE slightly inhibited LPS‐induced expression of IL‐6 protein (Fig. 2B), but significantly downregulated the mRNA expression of both IL‐6 and IL‐1β in the LPS‐treated macrophages (Fig. 2C). However, ABE could not attenuate the upregulation of TNF‐α mRNA and protein expression induced by LPS.

### 3.7 Effect of ABE on LPS‐induced iNOS and COX‐2 expression in raw 264.7 macrophages

**Figure 3:**
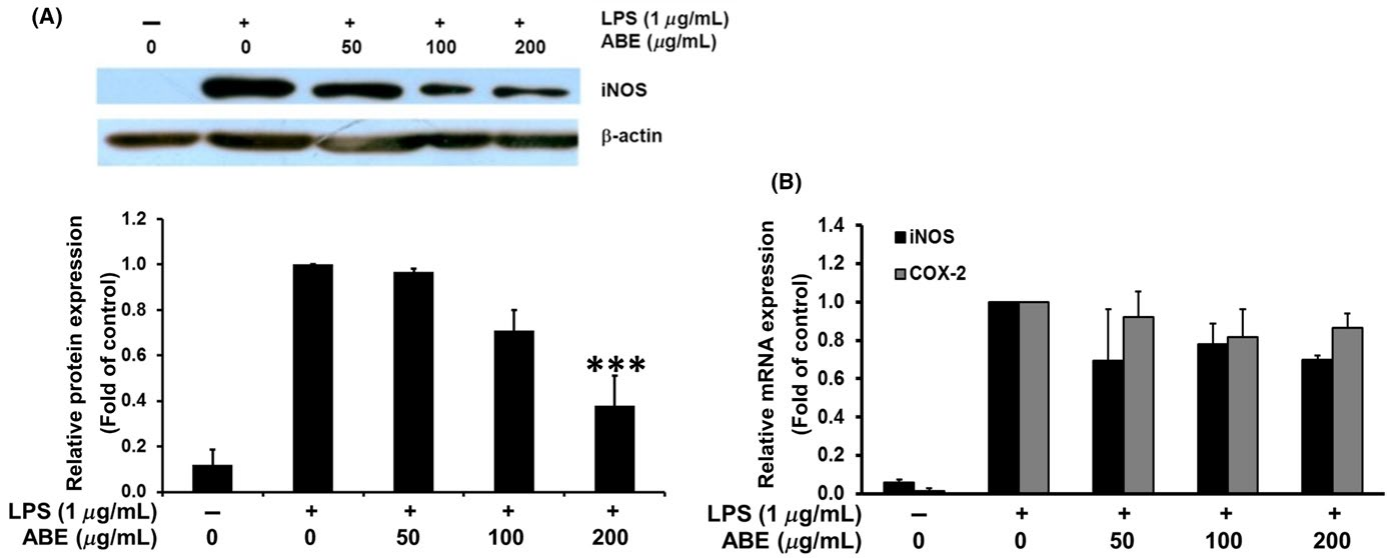
Effects of ABE on inducible nitric oxide synthase (iNOS) protein expression in LPS‐stimulated raw 264.7 cells (A) and on iNOS and COX‐2 mRNA expression (B). *** *p*<0.001 vs LPS‐treated group ABE, *Anoectochilus burmannicus* aqueous extract

NO production is catalyzed by iNOS enzyme whose expression can be upregulated in the LPS‐induced RAW macrophages. The inhibitory effect of ABE on LPS‐induced NO production as shown earlier might be due to the reduction in iNOS synthesis. The expressions of iNOS at both protein and transcript levels were then detected using immunoblotting and RT‐qPCR, respectively. The results found that ABE obviously decreases iNOS protein level (Fig. 3A), but slightly inhibits the LPS‐induced iNOS mRNA expression (Fig. 3B). Thus, the inhibitory effect of ABE on the LPS‐induced NO production is likely mediated by downregulation of iNOS expression.

Furthermore, the effect of ABE on expression of another inflammatory gene, COX‐2, was elucidated as shown in Figure 3B. ABE could slightly reduce COX‐2 mRNA expression about 20%. Alteration of COX‐2 expression may lead to the decreased production of prostaglandin E2 (PGE2), which also contributes to the progression of inflammation (Ricciotti & FitzGerald, 2011).

### 3.8 Effect of ABE on TNF‐α induced insulin‐resistant 3T3L1 adipocytes

**Figure 4:**
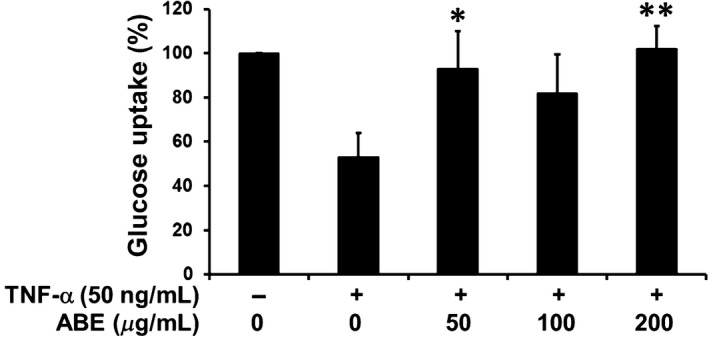
Anti‐insulin resistance activity of ABE in TNF‐α‐treated 3T3‐L1 adipocytes. * *p*<0.05, ** *p*<0.01 vs. TNF‐α‐treated group. ABE, *Anoectochilus burmannicus* aqueous extract

Insulin resistance in adipocytes has been shown to correlate with inflammatory response, especially TNF‐α leading to a low glucose uptake into the cells. ABE treatment significantly sensitized the insulin response in TNF‐α‐treated adipocytes as the glucose uptake level was 40% higher than that of the inflammation control (Fig. 4).

## 4 Discussion

In this study, we have firstly reported antioxidative stress, anti‐inflammatory, and anti‐insulin resistance activities of *A. burmannicus*. This plant has been commonly used in traditional Thai and Chinese medicines in form of decoction prepared by boiling herbs with water for 15–60 min. As to mimic the instruction for the use in daily life, we prepared the aqueous extract by boiling at 121°C for 30 min that following the method of Kim & Jang, (2011) with slight modifications. However, it is interesting whether the aqueous extract prepared by infusion is also biologically as effective as boiling since it would be further developed for consuming this plant as a hot tea.

Free radicals are potential inflammatory mediator contributing cellular or tissue damage, which is considered in the downregulation of inflammatory response (Lobo et al., 2010). ABE showed an efficiency to scavenge DPPH and ABTS free radicals in a dose‐dependent manner. Antioxidant activity of ABE might be involved with its anti‐inflammation and anti‐insulin resistance.

Macrophages play a significant role not only in host‐defense mechanisms but also inflammation. LPS‐stimulated macrophages would secrete several different inflammatory mediators, including IL‐1β, IL‐6, NO, and TNF‐α, (Esser et al., 2014; Schreckinger, Wang, Yousef, Lila, & Gonzalez de Mejia, 2010). The overproduction of these mediators has been related in several diseases caused by inflammation, such as obesity‐related insulin resistance (Xu et al., 2003), rheumatoid arthritis (Shrivastava et al., 2015), cancer (Chua, Chong, Liauw, Zhao, & Morris, 2012), atherosclerosis (Hamirani et al., 2014), and hepatitis (Connoy, Turner, & Nunez, 2011). Increasing in NO in the activated macrophages could induce a host‐defense mechanism and cellular or tissues damages leading to an inflammation (Ialenti, Ianaro, Moncada, & Di Rosa, 1992; Sampaio et al., 2013; Sharma, Al‐Omran, & Parvathy, 2007). Moreover, the level of NO has been used as a marker for the diagnosis and monitoring of response to anti‐inflammatory therapy (Zitt, 2005). Therefore, inhibition of NO could be a therapeutic approach toward inflammation and diseases caused by inflammation. Our results showed that ABE significantly inhibits the LPS‐induced NO production in macrophages.

Inducible NO synthase (iNOS), a key enzyme in NO production, is highly expressed during inflammation (Sharma et al., 2007). Several phenolic compounds, such as gallic acid, vanillic acid, coumaric acid, and ferulic acid, have been shown to directly inhibit the iNOS gene and protein expressions leading to the decreased level of NO production (Wang & Mazza, 2002). We found that ABE could suppress both mRNA and protein expressions of iNOS in LPS‐induced macrophages. These results suggested that ABE may attenuate NO synthesis via the downregulation of iNOS expression at the transcription and translation levels. However, ABE did not alter the protein expression of COX‐2; an inflammatory enzyme that catalyzes arachidonic acid into prostaglandins, which is inflammatory mediator and contributing inflammation (Ricciotti & FitzGerald, 2011). Several studies reported similar anti‐inflammation effects without the alteration of COX2. Kim, Hwang, & Park, (2014) showed chloroform layer of *Actinidia arguta* stems extract did not alter the COX‐2 level, but it strongly reduced NO production, proinflammatory cytokines in both protein and mRNA expression levels, and inhibited activation of NF‐κB and mitogen‐activated protein kinase (MAPKs). Likewise, Park, Kwon, & Sung, (2009) reported aloin could reduce NO production and level of proinflammatory cytokines, but had no effect on COX‐2 expression. Therefore, the reduction in NO production and IL‐1β level, but not COX‐2 expression by ABE might be sufficiently effective against inflammation.

Overproduction of IL‐1β, IL‐6, and TNF‐α has been implicated in several inflammatory diseases (Makki, Froguel, & Wolowczuk, 2013). IL‐1β and IL‐6 are important inflammatory cytokines secreted by macrophages. TNF‐α also is a major mediator in the development of chronic inflammation in diabetes mellitus and rheumatoid arthritis (Popa et al., 2007). Several studies reported suppressive effect of plant extracts on expression of these proinflammatory cytokines. For example, grape powder extract could reduce both inflammation and insulin resistance mediated by TNF‐α in adipocytes (Chuang et al., 2011). In addition, *Sasa borealis* leaves extract could improve insulin resistance by reducing cytokines secretion in obese C57/BL6J mice (Yang, Lim, & Heo, 2010). In our study, ABE could diminish the LPS‐induced expressions of IL‐1β and IL‐6, but was unable to inhibit either the elevated TNF‐α mRNA or protein expression in the activated macrophages. Similar to our results, lycopene (Feng, Ling, & Duan, 2010) and methanolic extract from edible mushrooms (Moro et al., 2012) inhibited the LPS‐induced production of IL‐6 and NO with decreased mRNAs of IL‐1β, IL‐6, iNOS, and NO without the effect on TNF‐α. Significantly, lycopene attenuated the effects of LPS by inhibiting a key inflammatory pathway related to mitogen‐activated protein kinase (MAPK), ERK1/2 and p38 MAPK, and NF‐κB, but not JNK (Feng et al., 2010). This observation supports the hypothesis that ERK1/2 predominantly triggers the expression of IL‐1β and IL‐6, whereas JNK is the main transduction pathway of TNF‐α, after LPS stimulation (Swantek, Cobb, & Geppert, 1997). It is possible that ABE may inhibit the LPS activation of MAPK and NF‐kB pathways leading to the decrease in IL‐1β and IL‐6.

Obesity and adipogenesis could increase production and secretion of proinflammatory cytokines such as TNF‐α, IL‐1β, and IL‐6 from macrophages into adipose tissues. These cytokines, especially TNF‐α, highly induce an insulin resistance in adipocytes in which the insulin signaling is interrupted resulting in an impaired glucose uptake (Makki et al., 2013; Nieto‐Vazquez et al., 2008; Rotter, Nagaev, & Smith, 2003). Although ABE had no effect on TNF‐α production in the LPS‐treated macrophages, we found that a low dose of ABE (50 μg/ml) could inhibit TNF‐α‐induced insulin resistance in adipocyte as shown by the increased glucose uptake. The anti‐insulin resistance of ABE could be directly useful for prevention and treatment of diabetes. Kinsennoside from *A. roxburghii* showed a significant hypoglycemic effect in streptozotocin‐induced diabetic rats. It ameliorated β‐cells’ damage caused by oxidative stress and NO, and also prevented weight loss in the diabetic animals (Zhang et al., 2007). In the study by Jian‐Gang Zhang (2015), polysaccharose isolated from *A. roxburghii* exerted antioxidant effect on diabetic mice leading to an improvement of glucose and lipid metabolism, an increase in immune protection, and a decrease in oxidative stress, which might contribute to its antidiabetic effect (Zhang et al., 2015).

The activities of ABE perhaps result from the presence of many phenolic compounds, namely, chlorogenic acid, coumaric acid, ferulic acid, and especially vanillic acid. These phenolic acids from other plants exert antioxidative stress (Cho et al., 2005; Cole et al., 2005; Huang et al., 2013; Srinivasan, Sudheer, & Menon, 2007), anti‐inflammation (C. & Wang, 2011; Cho et al., 2005; Hamalainen, Nieminen, Vuorela, Heinonen, & Moilanen, 2007; Huang et al., 2013; Rahman, Biswas, & Kirkham, 2006; Wang & Mazza, 2002), and anti‐insulin resistance (Peng et al., 2011). It is noteworthy that these phenolic acids found in ABE likely provide synergistic effects to scavenge the free radical and to inhibit both inflammation and insulin resistance. Nonetheless, the active compound(s) in ABE and their underlying mechanism of anti‐inflammation and anti‐insulin resistance need to be further investigated.

In summary, we have demonstrated antioxidation anti‐inflammation, anti‐insulin resistance, as well as safety of ABE. The anti‐insulin resistance effect of ABE in TNF‐α‐induced adipocyte could be due to the inhibition of macrophage and adipocyte inflammation. Significantly, ABE was not toxic as determined by *in vitro* cytotoxic and *in vivo* mutagenesis assays, suggesting its safety for further development and examination in animal and clinical models. Finally, our study provides scientific information for development and application of *A. burmannicus* as folk medicine, food ingredient, or food supplement for prevention of inflammation‐related chronic diseases. Further studies should be carried out to investigate the active components in ABE and to provide an insight into the underlying mechanisms of these effects.

## Funding Information

This work was funded by Faculty of Medicine Research Fund, Chiang Mai University, Chiang Mai, Thailand, the Plant Genetic Conservation Project under the Royal Initiative of Her Royal Highness Princess Maha Chakri Sirindhorn, Thailand, The National Research Council of Thailand (NRCT) and Grants‐in‐Aid from JSPS Core to‐Core Program, B. Asia‐Africa Science Platforms.

## Conflict of Interest

We have no conflict of interest to declare.
